# Supplementary material for: Universal radiation tolerant semiconductor
Source: Nat Commun. 2023 Aug 10;14:4855. doi: 10.1038/s41467-023-40588-0 (PMC10415340; doi:10.1038/s41467-023-40588-0)
Supplement: Supplementary file 1 — Supplementary Information [file 41467_2023_40588_MOESM1_ESM.pdf]

Supplementary Information

Universal radiation tolerant semiconductor

Alexander Azarov[1], Javier García Fernández[1], Junlei Zhao[2], Flyura Djurabekova[3], Huan He[3], Ru He[3], Øystein Prytz[1], Lasse Vines[1], Umutcan Bektas[4], Paul Chekhonin[4], Nico Klingner[4], Gregor Hlawacek[4] and Andrej Kuznetsov[1]

[1] University of Oslo, Centre for Materials Science and Nanotechnology, PO Box 1048 Blindern, N-0316 Oslo, Norway

[2] Department of Electrical and Electronic Engineering, Southern University of Science and Technology, Shenzhen 518055, China

[3] Department of Physics, University of Helsinki, P.O. Box 43, FI-00014 Helsinki, Finland

[4] Helmholtz-Zentrum Dresden-Rossendorf, D-01328 Dresden, Germany

Table of Contents

Supplementary note 1: dpa calculations and implant parameters normalization 2

Supplementary note 2: Explanation of the RBS/C data for extraction of relative disorder in β- and γ-Ga_2O_3 4

Supplementary note 3: Identification of the newly formed polymorph γ-Ga_2O_3 6

Supplementary note 4: Formation of metallic Ni precipitates 7

Supplementary note 5: Low mismatch interface stacking in γ/β-Ga_2O_3 double polymorph structures obtained by ion irradiation 8

Supplementary note 6: Defect balance at the β/γ-Ga_2O_3 interface 11

Supplementary note 7: Impact of Ga sub-lattice non-stoichiometry on the radiation tolerance in γ-Ga_2O_3 13

Supplementary note 8: Additional details of computational modelling 14

Supplementary References 20

Supplementary note 1: dpa calculations and implant parameters normalization

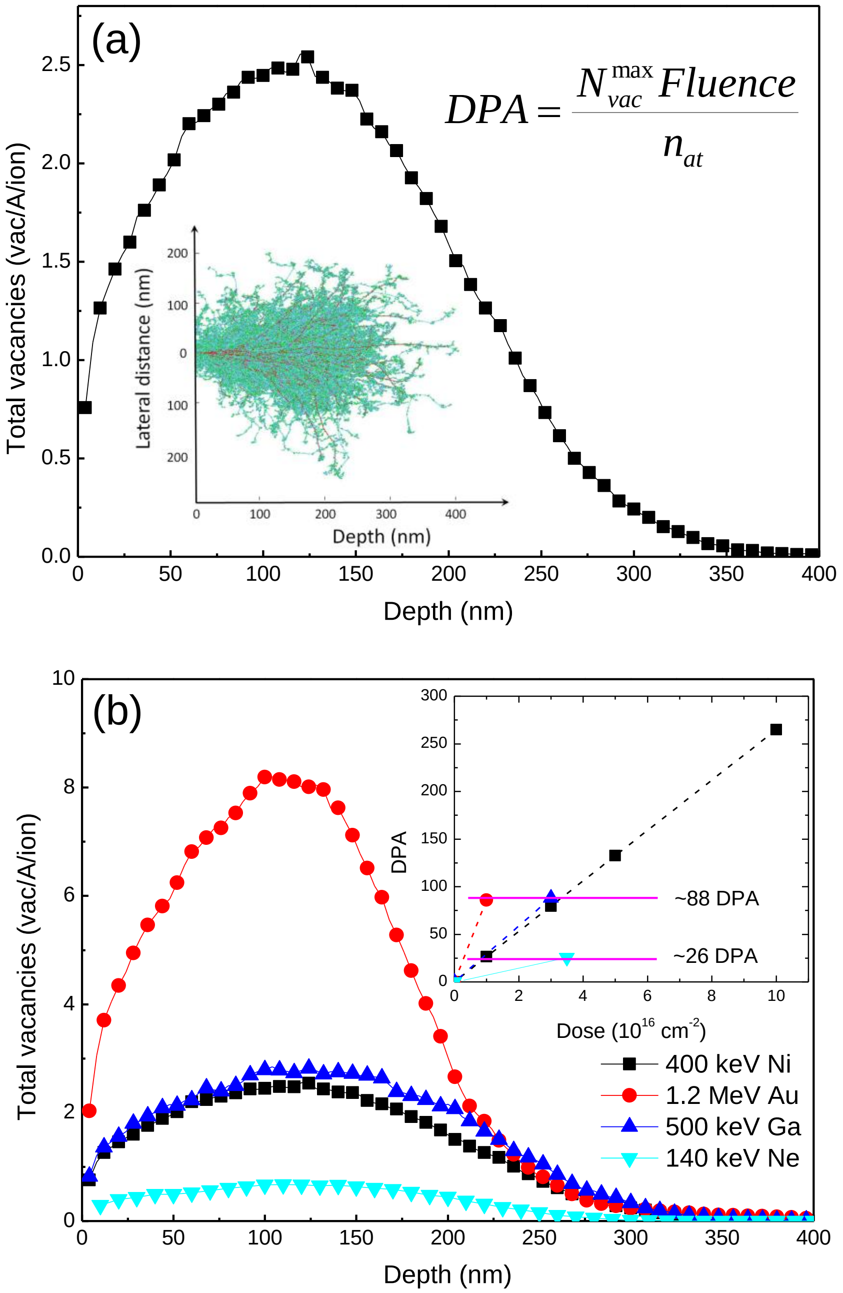

Supplementary Figure 1. (a) Schematic illustration of dpa calculation using SRIM. (b) Depth profiles of the total vacancies per implanted ion as calculated using SRIM code for Ne (down triangles), Ni (black squares), Ga (up triangles) and Au (circles) ions. The inset shows the dpa value taken in the maximum of the defect profiles as a function of the implanted fluence for the all implants used in the present study.

Here we use a conventional methodology of dpa calculation (see, for example, [1]. Supplementary Figure 1(a) illustrates the dpa calculation procedure. Initially, the depth profiles of ion generated vacancies were calculated using SRIM code [2] simulations in a full cascade mode as illustrated in Fig. 1(a) for 400 keV Ni implants. The inset in Supplementary Figure 1(a) shows a spatial distribution of primary defects (dots) obtained using the SRIM code simulations for 300 ion impacts. The SRIM calculations

were performed with 25 eV and 28 eV as the displacement energies for Ga and O atoms, respectively. The dpa values are taken from the depth corresponding to the highest magnitude in the SRIM vacancy generation profiles ($N_{\mathrm{vac}}^{\mathrm{max}}$) for a given fluence, normalized to an atomic density of β-Ga_2O_3 (n_{at} = 9.45×10^{22} at/cm^3) (see the equation in Supplementary Figure 1(a)). Note that the atomic density of γ-Ga_2O_3 is close to that of β-phase (n_{at} = 9.54×10^{22} at/cm^3) [3], so that the obtained dpa values can be used for both polymorphs. It should be noted that there are no experimental values of the displacement energies in Ga_2O_3 polymorphs. Even for the most intensively studied β-phase, there is only one recent theoretical paper where the authors determined the displacement energies to be 28 and 14 eV for Ga and O sublattices, respectively [4]. According to the calculations, the lower values of the displacement energies results in the higher concentration of the primary defects by a factor of 1.55 as compared to those done with SRIM default values of displacement energies. Thus, the data obtained with the SRIM default values can be considered as a lower limit of the dpa thresholds. Thus, in the present paper the dpa calculations are used to normalize the irradiation conditions and to compare the results obtained for different ions.

The implantation parameters of Ne, Ga and Au ions were adjusted in such a way that they resulted in similar absolute numbers and distributions of primary defects. In order to illustrate this normalization, the SRIM calculated depth profiles of total generated lattice vacancies as a function of depth are plotted in Fig. 1(b) for all four ions studied. It is seen that all the defect generation profiles have a similar shape with the R_{pd} is in the range of 110-120 nm (see also Table 1 in the main text). It is seen that Ga and Ni ions produce nearly the same number of primary defects since the atomic masses of these atoms are close to each other. In its turn, Au ions generate denser collision cascades containing more defects, as compared to both Ga and Ni ions, by a factor of 3, while Ne ions generate less defects as compared by Ni ones by a factor of 3.5. The inset in Supplementary Figure 1(b) shows the calculated dpa values as a function of the fluence for all four ions used in the present study. The solid lines in the inset indicates the implantation fluences for all the ions corresponding to the similar dpa values of ~88 and ~26, which were used for the additional Ne, Ga and Au implantations.

Supplementary note 2: Explanation of the RBS/C data for extraction of relative disorder in β- and γ-Ga_2O_3

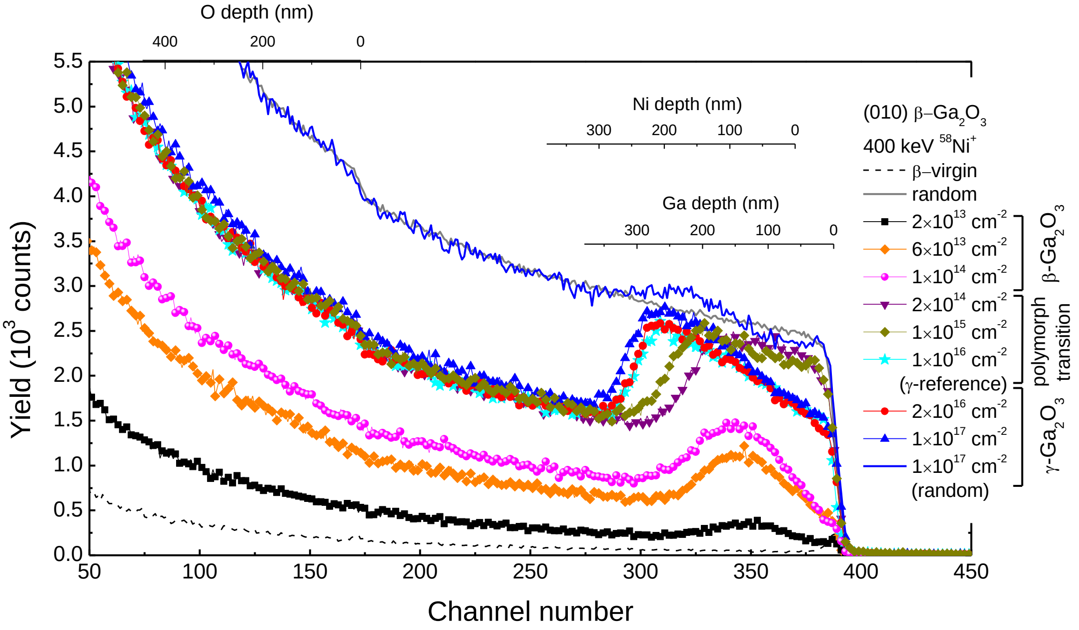

Supplementary Figure 2. RBS/C spectra of (010) β-Ga_2O_3 samples implanted with 400 keV $^{58}Ni^+$ ions for different fluences as indicated in the legend. The channeling spectrum of the unimplanted (β-virgin) sample is shown by a dashed line for a comparison. Spectra acquired for the random incidence of the He beam are shown too by thick lines.

Supplementary Figure 2 shows full-scale RBS/C spectra of Ni implanted β-Ga_2O_3 samples for different fluences. The labeled Ga, Ni and O depth scales are included in the figure with the surface position corresponding to channel numbers at 386, 370 and 172, respectively. It should be noted that the sensitivity of the RBS technique depends on the atomic mass of the elements comprising the target, so that it is more sensitive to Ga atoms as compared to O atoms. Thus, only the Ga parts of the RBS/C data are used and discussed below as it is also done in the main text.

As seen from Supplementary Figure 2 for the low fluences (2×10^{13}-1×10^{14} Ni/cm^2) the RBS/C spectra form a well-defined disorder peak visible on the Ga sublattice centered around the maximum of the nuclear energy loss profile - R_{pd} (see Table 1). The increase of the fluence up to 2×10^{14} Ni/cm^2 leads to the formation of the "box-like" disorder profile extending up to 180 nm from the surface. The disorder level in this layer does not reach the random level confirming that β-Ga_2O_3 is not fully amorphous state. Further fluence increase up to 1×10^{15} Ni/cm^2 leads to the broadening of this "box-like" disorder profile into the bulk of the sample with nearly maintained amplitude of the disorder level. Prominently, the increase of the fluence to 1×10^{16} Ni/cm^2 leads to further broadening into the bulk and a remarkable decrease in the RBS/C yield of the feature

we called “box-like disorder” above. Previously, similar high level disorder saturation in (010) β-Ga_2O_3 was observed for room temperature implants and the authors suggested that such behavior can be attributed to the phase transitions; however, without specific characterization/interpretation attempts [5]. It its turn, Lorenz *et al.* [6] reported near the surface amorphization in Eu implanted (-201) β-Ga_2O_3 that can be attributed to the Eu-induced defect stabilization or poor channeling conditions due to β/γ as shown in Supplementary note 5. Recently, Azarov *et. al.* [7] demonstrated that this evolution is attributed to the disorder-induced phase transformations in the implanted region, so that a double polymorphic structure forms. Following Ref. [7], we associate the (2×10^{14}-1×10^{16} Ni/cm^2) fluence range to the polymorph transition (see “polymorph transition” label in Supplementary Figure 2 and the arrow in Fig. 1(d)).

Notably, even though early works [7,8] identified this new polymorph as kappa-phase, now, it is established that it maintains γ-phase symmetry, independently of the ion type used in the implants [9,10], fully consistently with our observations/interpretations in this work. Moreover, according to the literature [7,9,10] and the STEM data in the present work (Supplementary notes 3 and 7) this γ-Ga_2O_3 exhibits high crystallinity. Thus, for Fig. 1 and Supplementary Figure 2 we consider the γ-Ga_2O_3 part of the double γ/β polymorph structure formed with 1×10^{16} Ni/cm^2, as a “reference γ-Ga_2O_3” to which we may interrelate disorder changes eventually occurring for higher fluences. Notably, the absolute RBS/C yield for this “reference γ-Ga_2O_3” spectrum is significantly higher than that for the initial “virgin β-Ga_2O_3”. This is not a consequence of a poor crystallinity. It is because the alignment for the experiments in Fig. 1 and Supplementary Figure 2 was done along the (010) direction in the β-Ga_2O_3, which is not main channel in γ-Ga_2O_3, according to the orientation relationship at the γ/β interface as measured in Supplementary note 5.

Thus, for Fig. 1(d) we calculate the relative disorder in β-Ga_2O_3 implanted with low fluences (2×10^{13}-1×10^{14} Ni/cm^2) using one of the conventional algorithms [22] obtaining the data ranging from 0 to 1 corresponding to the perfect and amorphous materials, respectively. In its turn, the relative disorder in γ-Ga_2O_3, eventually happening in the course of the high fluence accumulation, can be estimated by the deviations of the corresponding RBS/C amplitudes from that we defined as “reference γ-Ga_2O_3” i.e. in the sample fabricated with 1×10^{16} Ni/cm^2 implants. Accounting that there are practically no deviations in the RBS/C yields for the high fluence implanted

samples as compared to that in the reference γ-Ga_2O_3, we set the relative disorder in the γ-Ga_2O_3 at zero for all high fluences used, see Fig. 1(d) in the main text. Thus, we refer to the RBS/C spectrum obtained upon 1×10^{16} Ni/cm^2 implants as having the "characteristic shape" to interrelate the rest of the data in Fig. 1.

Supplementary note 3: Identification of the newly formed polymorph γ-Ga_2O_3

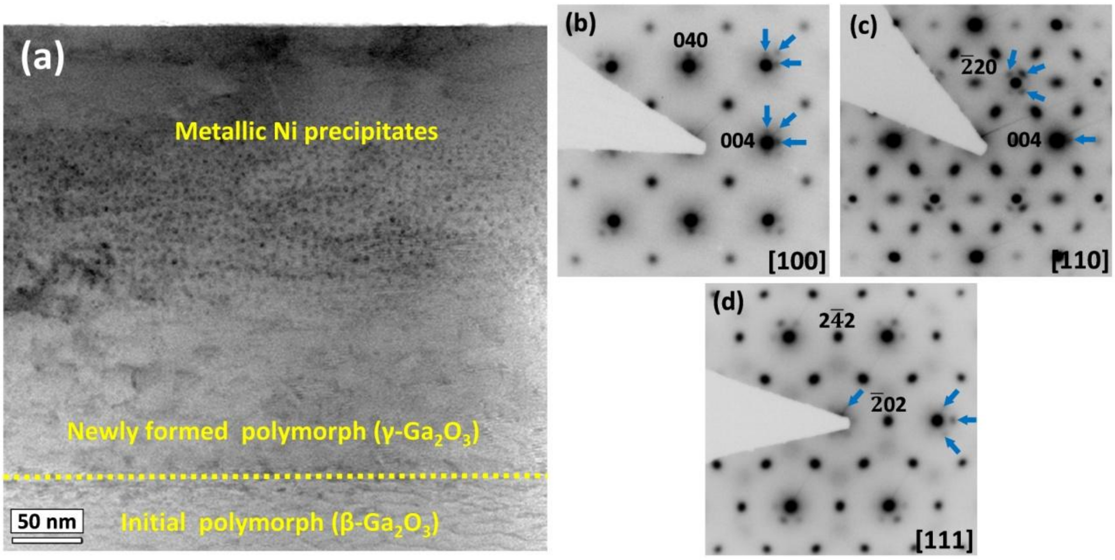

Supplementary Figure 3. (a) Low magnification BF-STEM image of the 1×10^{17} Ni/cm^2 implanted sample together with (b-d) the SAED patterns from the new polymorph layer outside of Ni-precipitation zone. The indexing is according to γ-Ga_2O_3 along the zone axes (b) [100], (c) [110], and (d) [111]. The blue arrows indicate the extra spots described in the text.

Supplementary Figure 3(a) show the low magnification BF-STEM images corresponding to the sample implanted with 1×10^{17} Ni/cm^2. Supplementary Figures 3(b-d) show the SAED diagrams indexed according to the γ-Ga_2O_3 polymorph (space group *Fd-3m*) along the zone axes [100], [110] and [111], respectively. Nevertheless, new findings are observed in the SAED diagrams: the presence of extra weak spots around the family of planes {400}, {440}, {222} and the (000), indicated with a blue arrow, which cannot be indexed according to the cubic γ-Ga_2O_3 polymorph. The cause of these extra spots is due to the presence of cubic metallic Ni (fcc, space group *Fm-3m*) and a double diffraction effect as a result of the coherence between cubic γ-Ga_2O_3 and cubic Ni metallic precipitates. The diffraction spots related to the metallic Ni are marked in green while the double diffraction spots are indicated with a pink arrow in Fig. 1(c). Similar double

diffraction reflections have been reported previously in literature for cubic precipitates inside different matrices [12,13].

Supplementary note 4: Formation of metallic Ni precipitates

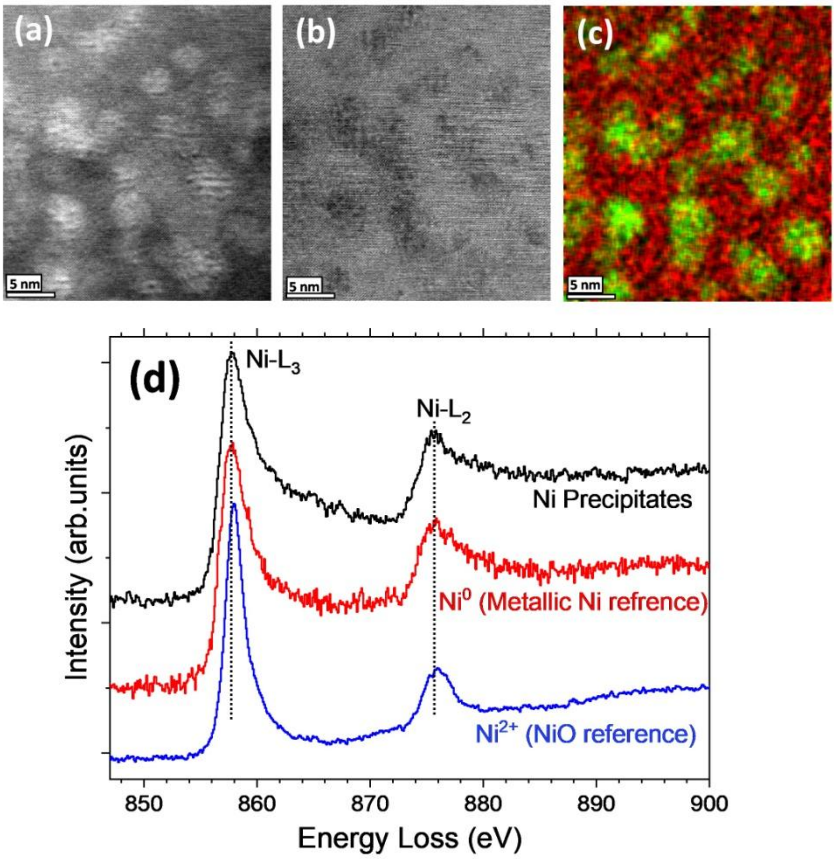

Supplementary Figure 4. High Magnification (a) ADF-STEM and (b) BF-STEM of the 1×10^{17} Ni/cm^2 sample. (c) Corresponding STEM- EDS map acquired for Ga (red) and Ni (green). (d) STEM-EELS Ni L_3 and L_2 edges obtained from the Ni precipitates (black line), metallic Ni reference (red line) and NiO reference (blue line).

The presence of Ni precipitates inside γ-Ga_2O_3 layer is confirmed by EDS mapping. Supplementary Figure 4(a-b) show the ADF and the BF-STEM images at higher magnification revealing the precipitates, respectively. The corresponding EDS map using the Ga-Kα line in red together with the Ni-Kα in green is shown in Supplementary Figure 4(c). In order to know more about the oxidation state, we have recorded an EELS spectrum corresponding to the $L_{3,2}$ line of Ni precipitates, Supplementary Figure 4(d), confirming that the precipitates are mainly formed by metallic Ni and not by NiO. A similar line shape has been found before in other articles corresponding to metallic Ni [14,15]. Furthermore, for a better comparison, the $L_{3,2}$ Ni line profile of two references corresponding to metallic Ni and NiO are shown as well. The L_3/L_2 ratios obtained were

as follows: 1.5, 1.4 and 2.8 for the Ni precipitates, metallic Ni reference and NiO reference, respectively. The good agreement between the stabilized metallic Ni precipitates as a consequence of the implantation and the metallic Ni reference is clear.

Supplementary note 5: Low mismatch interface stacking in γ/β-Ga_2O_3 double polymorph structures obtained by ion irradiation

Independently of the type of ions used in the present study we observed the formation of the same stacking at the γ/β-Ga_2O_3 double polymorph structures obtained by ion irradiation. This is illustrated below by applying two different methods for the stacking orientation determinations: STEM and EBSD. For example, in the samples implanted by Au, the orientation relationship between two polymorphs founded in the STEM is γ [100]//β [201], γ [110]//β [132] and γ [112]//β [102]. Supplementary Figure 5(a-c) shows schematic representation of γ-Ga_2O_3 (ICSD: 236276 [16]) and β-Ga_2O_3 (ICSD: 83645 [17]) along the interface together with the corresponding diffraction patterns, for the three mentioned orientation relationships.

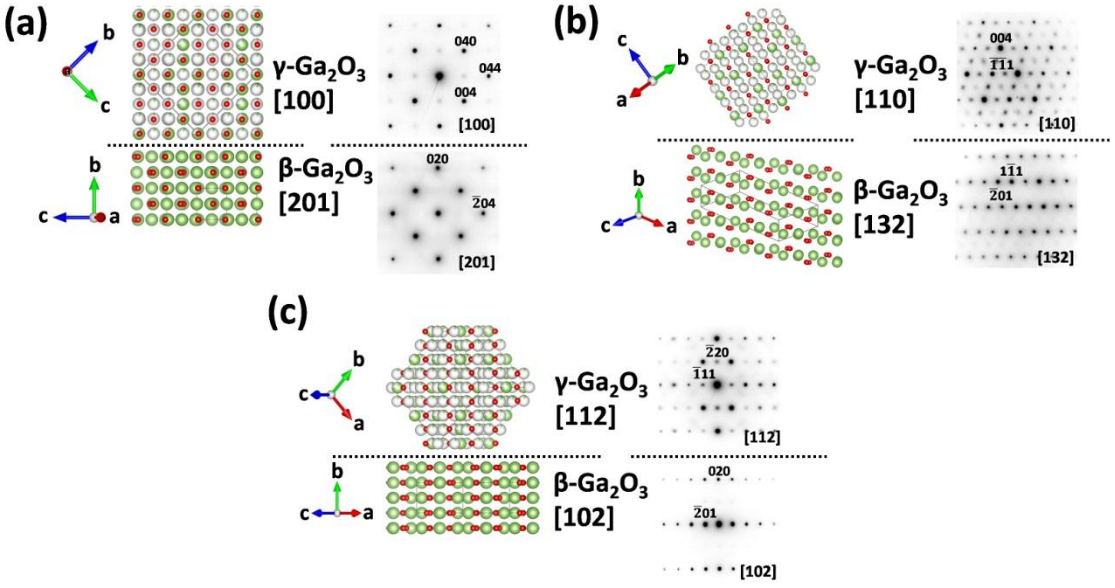

Supplementary Figure 5. Schematic representation of: (a) γ-Ga_2O_3 [100]// β-Ga_2O_3 [201], (b) γ [110]//β [132], (c) γ [112]//β [102] along the interface together with the corresponding diffraction patterns. Color code: Ga (green) O (red). The interface is represented with a dashed black line.

Interestingly, the lattice mismatch for the stacking in Supplementary Figure 5 is quite similar for each zone axis, as summarized in Supplementary Table 1. This may facilitate the initial β-to-γ transformation as well as the conversion of additional parts of the β-substrate into the γ-film during further accumulation of the fluence.

Orientation relationship	Experimental Interplanar distances (Å)
γ [100]//β [201]	γ-Ga_2O_3 $d_{(044)}$ = 1.47 ± 0.03
	β-Ga_2O_3 $d_{(-204)}$ = 1.46 ± 0.04
γ [110]//β [132]	γ-Ga_2O_3 $d_{(-111)}$ = 4.83 ± 0.03
	β-Ga_2O_3 $d_{(-201)}$ = 4.92 ± 0.02
γ [112]//β [102]	γ-Ga_2O_3 $d_{(-1-11)}$ = 4.78 ± 0.02
	β-Ga_2O_3 $d_{(-201)}$ = 4.82 ± 0.03

Supplementary Table 1. Summary of experimental interplanar distances measured from SAED

For comparison, we also used EBSD on the γ/β-Ga_2O_3 double polymorph structures fabricated with Ne implants. For these measurements, a part of the sample was masked during the irradiation, thus EBSD was performed on both, the unirradiated surface section as well as on the Ne irradiated surface. The mounting of the sample was done so that the β-Ga_2O_3 <010> lattice direction is aligned parallel to the Z-axis of the SEM reference system, while the β-Ga_2O_3 <102> lattice direction is aligned parallel to the Y-axis of the SEM reference system. Notably, since both the irradiated and unirradiated parts of the sample underwent exactly the same alignment, the orientation relationship between the unirradiated β-Ga_2O_3 phase and the phase formed after irradiation was only affected by the random error of the EBSD system, which is typically ≤1° in terms of the misorientation [19].

Firstly, we found that EBSD patterns recorded in the unirradiated and Ne-irradiated areas of the sample matched with the β- Ga_2O_3 and the γ- Ga_2O_3 polymorph signatures, as illustrated in Supplementary Figures 6 (a) and (b), respectively.

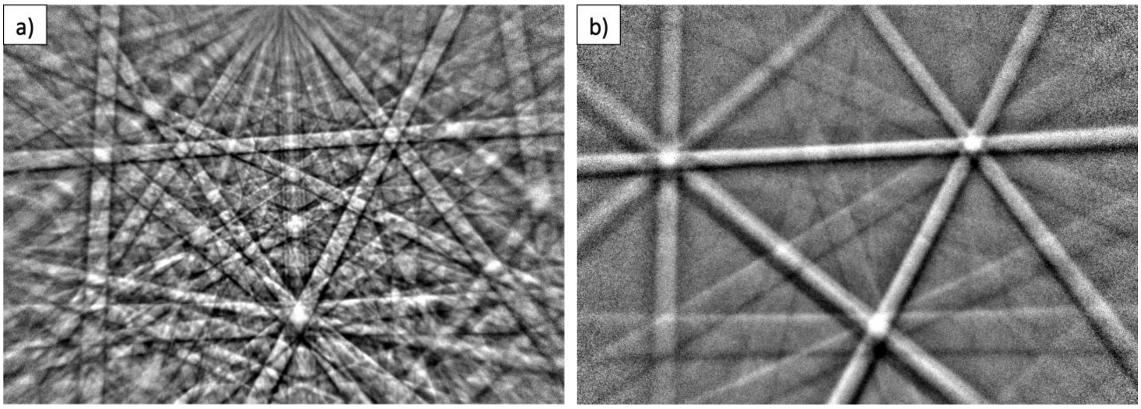

Supplementary Figure 6. Recorded EBSD patterns from (a) initial β- Ga_2O_3 and (b) γ- Ga_2O_3 polymorph after Ne irradiation.

The pristine β-Ga_2O_3 crystal gives a high quality EBSD pattern, where many high indexed Kikuchi bands and details inside those are discernible. In comparison, the EBSD pattern quality from the γ-Ga_2O_3 area exhibits less contrast, only low indexed strong intensity bands are clearly distinguishable. A lower EBSD pattern quality may be explained by the intrinsic defective spinel structure of γ-Ga_2O_3 [20] and not necessarily by addition defects introduced as a consequence of irradiation. However, a quantification from this pattern is not possible. Furthermore, it should be pointed out that EBSD is a surface sensitive method, the depth of the surface layer contributing to the EBSD pattern is of order 10 nm [21].

Regarding the absolute crystal orientation, the Bruker EBSD software provided the following Euler angles:

β-Ga_2O_3: (309.9°; 89.5°; 358.9°)

γ-Ga_2O_3: (309.9°; 43.6°; 89.0°)

Using these Euler angles an orientation relationship between both phases was calculated to:

β-Ga_2O_3 [100] // γ-Ga_2O_3 [1 -5.6 -1]

β-Ga_2O_3 [010] // γ-Ga_2O_3 [1 0 1]

β-Ga_2O_3 [001] // γ-Ga_2O_3 [-1 0 1]

and

γ-Ga_2O_3 [100] // β-Ga_2O_3 [0 1.9 -1]

γ-Ga_2O_3 [010] // β-Ga_2O_3 [-1.9 -0.1 -1]

γ-Ga_2O_3 [001] // β-Ga_2O_3 [0 2 1]

The fractional numbers indicate that there is misalignment between some low indexed directions of the β-Ga_2O_3 and the γ-Ga_2O_3 polymorphs. This orientation relationship is consistent with results in Supplementary Figure 6 obtained by SAED TEM, which indicates that (*i*) interface stacking in γ/β-Ga_2O_3 double polymorph structures is independent on the ion type used in fabrication process and (*ii*) it is verified by two different methods.

Supplementary note 6: Defect balance at the β/γ-Ga_2O_3 interface

Fig. 1(a) indicates an increase in thickness of the newly formed γ-Ga_2O_3 layer as a function of the ion fluence. Our interpretation is that there is a disorder threshold for the β-to-γ transition. Thus, the thickness increase of the γ-polymorph in Fig. 1(a) might be a function of the balance between defect generation and annihilation rates at the β/γ-Ga_2O_3 interface. The defect generation rate is a function of the incident beam particles mass, energy and flux. Importantly, all these parameters were kept constant for the data in Fig. 1(a). Otherwise, the amount of the generated defects might obviously scale with the fluence. Therefore, we may try to estimate the dpa threshold required for the polymorph conversion using the SRIM data presented in Supplementary Figure 1 and plotting the DPA values at the β/γ interface as a function of the fluence. The results of this analysis are shown in Supplementary Figure 7. Interestingly, as seen from Supplementary Figure 7, the dpa dependence of the β-to-γ transition as measured at the β/γ interface is not constant, as could be expected for a threshold. Instead, it exhibits an increasing trend while the β/γ interface advances deeper into the bulk. It implies that in Supplementary Figure 7 we haven't accounted for additional factors governing the defect balance in Fig. 1(a). Indeed, as mentioned above, the residual defect concentration is a balance between the defect generation and annihilation processes. However, as know from literature, the defect annihilation rates are primarily affected by the irradiation temperature [7,11]. Thus, accounting that the data in Fig. 1(a) were collected at the same temperature, in the first approximation, the assumption of similar annihilation rates in samples implanted with different fluences seems to be reasonable (even though we used different beam exposure times which may have affected the diffusion-controlled annihilation). Nevertheless, there should be a reason for the distinct increase of the dpa threshold with an increase of the fluence and, therefore, advancing the interface deeper in the bulk in Supplementary Figure 5. Notably, as seen from the inset in Supplementary Figure 7, the defect generation rate at the depth of the β/γ interface rapidly decreases with its advance into the bulk of the sample, meaning significant differences for the density of the collision cascades in the samples, even though the "nominal" dpa scales as a function of fluence. We believe these cascade density effects – unaccounted in the analysis in Supplementary Figure 7 are responsible for the non-constant dpa threshold. This conclusion is also supported by the recent results on the role of the density of collision cascades on the defect formation in β-Ga_2O_3 [18]. Concurrently, we cannot exclude that the defect balance at the β/γ interface

can be affected by the defects diffusion too, altogether, leaving room for further studies paved by our interpretations.

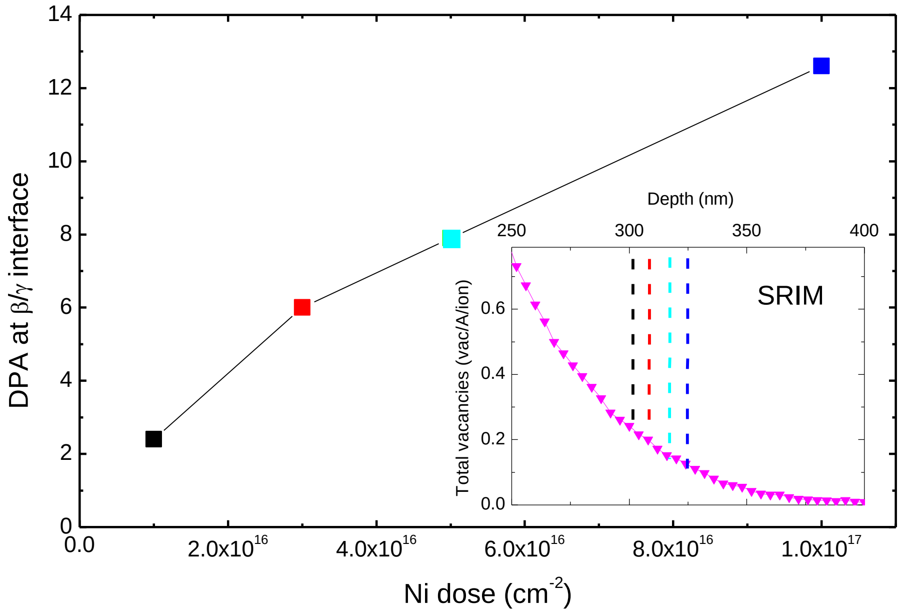

Supplementary Figure 7. dpa values calculated at the β/γ interface as a function of the ion fluence for the 400 keV Ni irradiations. The inset depicts a part of the SRIM predicted defect generation profile at the depth corresponding to the β/γ interface, using the same color code for the β/γ interface position as that in Fig. 1(a) (black - 1×10^{16}, red - 3×10^{16}, cyan - 5×10^{16}, and blue -1×10^{17} Ni/cm^2).

Supplementary note 7: Impact of Ga sub-lattice non-stoichiometry on the radiation tolerance in γ-Ga_2O_3

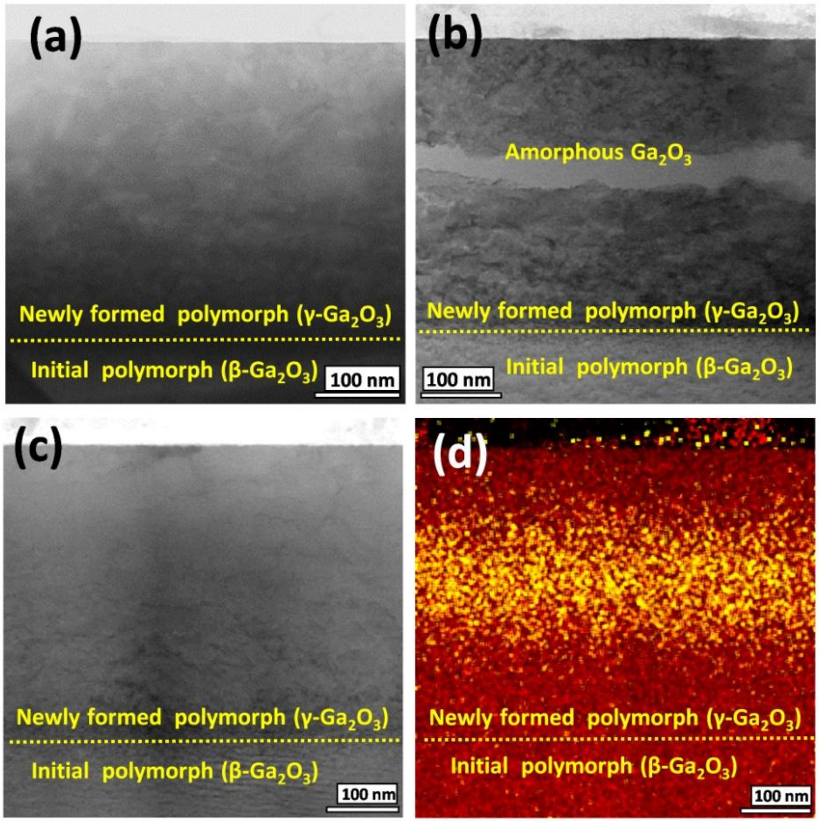

Supplementary Figure 8. Low magnification BF-STEM images and EDS maps of Ga and Au implanted γ-Ga_2O_3; (a, b, c) 1×10^{16} Ga/cm^2, 3×10^{16} Ga/cm^2 and 1×10^{16} Au/cm^2 samples, respectively; (d) EDS elemental maps of the 1×10^{16} Au/cm^2 sample.

Supplementary Figure 8 is complimentary to Fig.2 in the main text. As seen from Supplementary Figures 8(a) and (b) there is a Ga content threshold for a deviation from the radiation tolerance due to chemical effects. Indeed, three-fold change in the Ga content, corresponding to the fluence increase from 1×10^{16} to 3×10^{16} and Ga/cm^2, implies a dramatic difference in the radiation tolerance behavior, literally the intact crystallinity turns into the amorphization of the middle part of the γ-Ga_2O_3 film, compare Supplementary Figures 8(a) and (b). Otherwise, γ-Ga_2O_3 easily tolerates the dpa corresponding to that in Supplementary Figure 8(b), if created by other ions, e.g., Au – see Supplementary Figure 8(c). We attribute this effect to the increasing deviations from the γ-Ga_2O_3 stoichiometry in Ga sublattice, which is also consistent with the theoretical investigations of the chemical roles of different impurities in Fig. 4 of the main text. Meanwhile the elemental distribution of Au is

illustrated by the EDS map in Supplementary Figure 8(d), using the Ga-Kα (red) and Au-Lα (yellow) lines.

Supplementary note 8: Additional details of computational modelling

As shown in Supplementary Figures 9(a-b), a detailed comparison of the RDFs and pairwise PRDFs of the pristine β- and γ-Ga_2O_3 lattices reveals that the significant difference lies in the Ga sublattice with the Pearson correlation coefficient, Pr = 0.762 (see the Method part of the main text for details). Both the O sublattices of the β- and γ-Ga_2O_3 follow the close-packed face-centered cubic (fcc) stacking, whereas the Ga atoms occupy the tetrahedral and octahedral sites in different symmetries, as shown in Supplementary Figure 9(c).

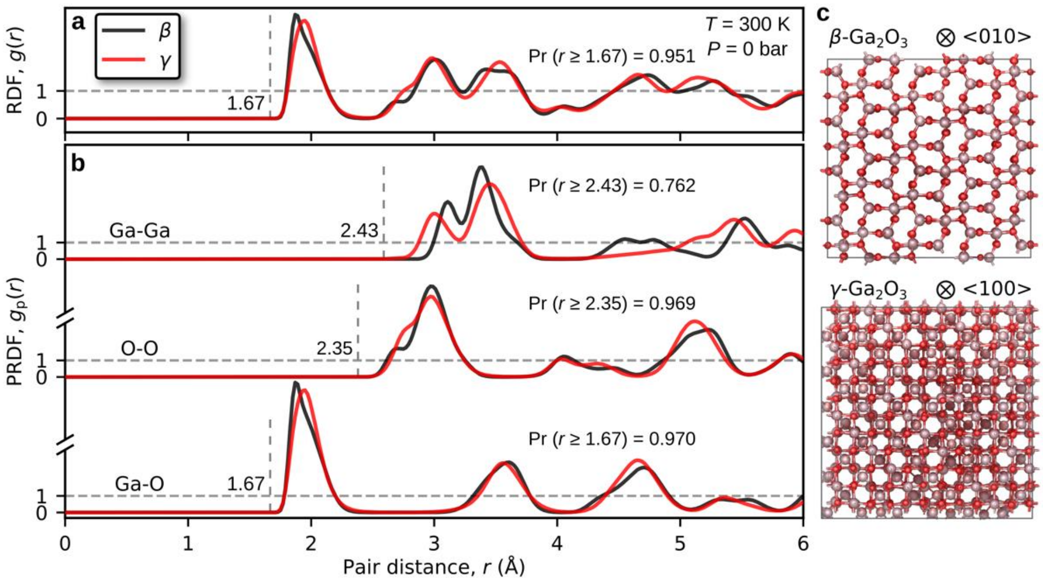

Supplementary Figure 9. (a) Total radial distribution functions (RDFs) and (b) pairwise partial RDFs (PRDFs) of the pristine β- (black lines) and γ-Ga_2O_3 (red lines) lattices at 300 K and 0 bar. The Pearson correlation coefficients, Pr, between the non-zero parts (ranges from the vertical dashed lines up to 6.0 Å) of the RDF and PRDF curves are shown, revealing that the major difference between the β- and γ-Ga_2O_3 lattices are the configuration of Ga sublattice with rather low Pr of 0.762. (c) Illustration of the pristine β- and γ-Ga_2O_3 supercell with brown Ga and red O atoms

As shown in Supplementary Figure 10(a), a further analysis of the 1st and 2nd Ga-Ga shells indicate a high similarity between the two 1st Ga-Ga shells (Pr = 0.830), however, completely uncorrelated 2nd Ga-Ga shells are seen, as the peaks in the β-Ga PRDFs are vanished in the γ-Ga PRDFs (Pr = 0.037). The 1st- and 2nd-shell ordering of the β-Ga sublattice is illustrated in Supplementary Figure 10(b-c). In the 1st shell, both the 4-fold

(tetrahedral) and 6-fold (octahedral) Ga atoms have 11 Ga neighbors as shown in the zoomed local bonding networks. In the 2nd shell, 4-fold Ga has 8 Ga neighbors, and 6-fold Ga has 6. As a matter of fact, the specific pair between 6-fold Ga and the 6-fold Ga neighbors (shown as the yellow and orange bonds in Supplementary Figure 10(c)) contribute to the shallow peak in the *β*-Ga PRDF around the 4.75 Å (Supplementary Figure 10(a)).

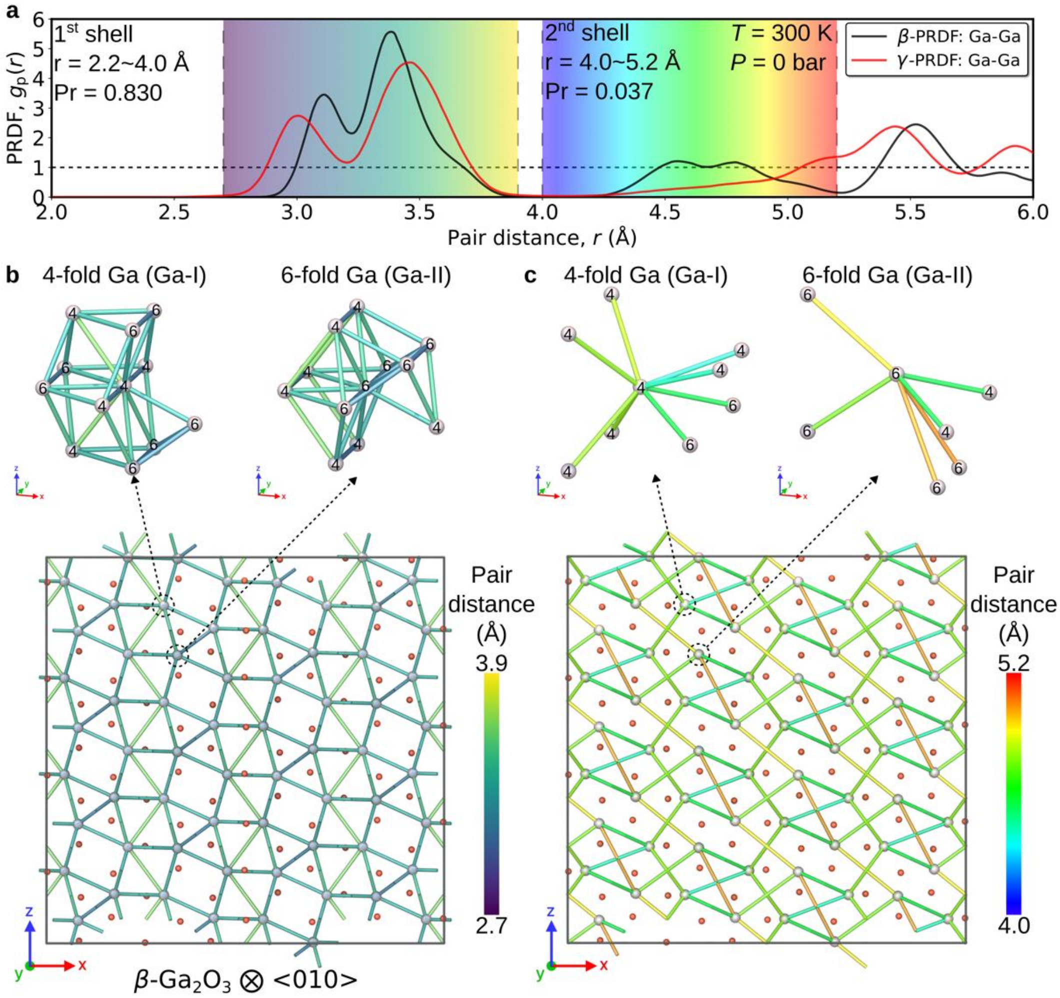

Supplementary Figure 10. (a) Ga-Ga PRDFs of the pristine β- (black line) and γ-Ga_2O_3 (red line) lattices at 300 K and 0 bar with the marked 1st and 2nd Ga-Ga shells of 2.2~4.0 Å and 4.0~5.2 Å, respectively. The range-specified Pr values indicate a high similarity in the 1st Ga-Ga shells of the pristine β- and γ-Ga_2O_3 lattices, whereas a significant difference in the 2nd shells. The two color-coded shadow regions correspond to the two Ga-Ga pair matrices in the β-Ga_2O_3 with visualized bonds, as shown in (b). This 1st-shell short-range ordering of the β-Ga sublattice is randomized in the γ-Ga_2O_3 phase, whereas the 2nd-shell ordering is completely missing.

Supplementary Figure 11 illustrates the detailed snapshots for every 100-FPs step both in β and γ phases. As seen from Supplementary Figure 11, the O-sublattice retains the fcc stacking perfectly. The β-Ga sublattice gradually transfers to γ-like configuration, whereas no significant change is detected in the γ-Ga sublattice.

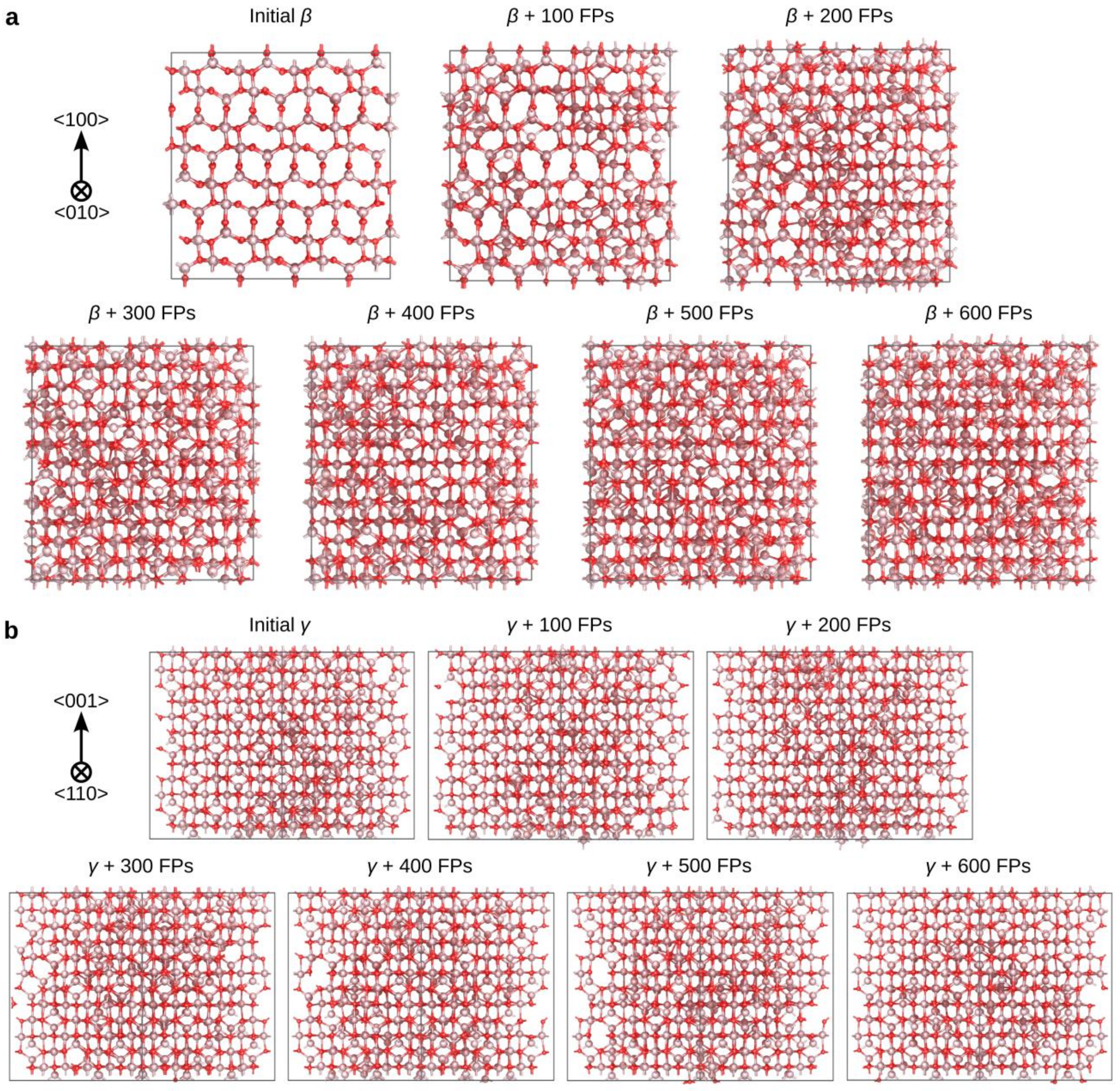

Supplementary Figure 11. The snapshots show modifications of the (a) β-Ga_2O_3 and (b) γ-Ga_2O_3 from the pristine lattices up to the lattices with added 600 Frenkel pairs. Ga ions are shown in brown and O in red. Note that the view angle of the γ-Ga_2O_3 here is turned to <110> (45° turned from <100> in Fig. 3(b) of the main text), to emphasize the same symmetry of the β/γ-O sublattices.

As shown in Supplementary Figure 12, the analysis of the Pearson correlation coefficient reveals the evolution of the structural properties of the Ga sublattices with different numbers of FPs generated. It can be clearly seen that the 1st-shell curves preserve a high similarity (Pr > 0.800) for both the β- and γ-Ga_2O_3 owing to the high similarity between initial β/γ curves (Pr = 0.830, Supplementary Figure 10(a)). On the other hand, the generated FPs have only a marginal effect on the 2nd-shell γ-Ga curves, however, imply dramatic changes onto the 2nd-shell β-Ga curves, indicating a distinct structural transition causing by the cumulated Ga FPs in the β lattice.

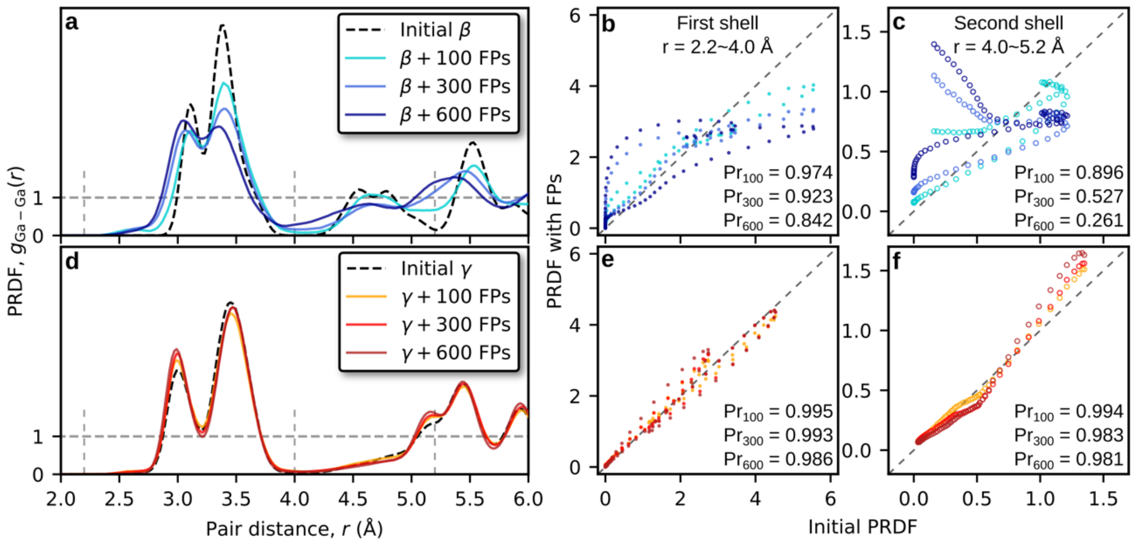

Supplementary Figure 12. Detailed Pr analysis: (a, d) The Ga-Ga PRDFs of the β- and γ-Ga_2O_3 lattices after different numbers of FP iterations. The initial β/γ curves are shown as dashed lines for reference. (b-c, e-f) The 1st- and 2nd-shell curves are plotted against the corresponding initial curves. The high similarity of the 1st-shell curves retains after 600 FP iterations, whereas the β-Ga 2nd-shell curves change significantly upon the presenting Ga FPs, and eventually transfer to more γ-like (Fig. 3c-d in the main text).

In Supplementary Figure 13, we analyze the stability of the fcc O sublattice during collision cascades. Supplementary Figure 13a shows the mean square displacement (MSD) for all Ga and O atoms displaced during the cascades. By increasing the threshold displacement (the minimal displacement distance that is counted in the calculation of MSD) we reveal stronger mobility of O ions during the cascades. The red curve that plots the MSD values for O atoms is much higher that the black curve, indicating that O atoms were displaced in cascades much more efficiently. This is also seen in the exemplary snapshots at different time instances of the cascade evolution (0, 0.2, 2.5 and 50 ps) that reveal formation of large amount of coordination defects during the cascade, which practically fully disappear in the O sublattice leaving behind only very few vacancy clusters. Given long time between the ion impacts in standard ion accelerators, these remaining defects are expected to recover further given the strength of the O sublattice, which we observe in our simulations.

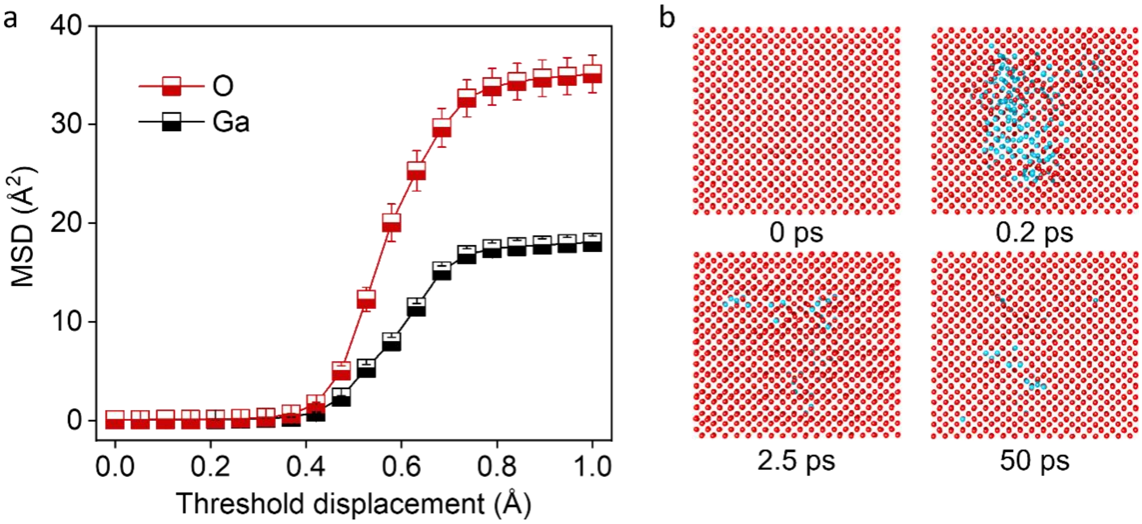

Supplementary Figure 13. (a) Mean square displacement (MSD) of the Ga (black squares) and O (red squares) ions during the collision cascades calculated from the comparison of the ion positions in the first and the last (50 ps) frames after the cascade. The MSD is plotted as a function of the threshold displacement, therefore, only atoms with the final displacement larger than the threshold value are included in the statistics. The error bars are the standard error of the mean collected in 120 MD simulations. (b) The snapshots showing the evolution of the fcc O sublattice at different time instances during an exemplary cascade case. The oxygen ions are colored according to the coordination number (the red color is used for the ions with Z = 11 (with one neighboring vacancy) or 12 (perfect fcc), to exclude coloring of atoms around single vacancies, since relaxation of such defects causes only insignificant distortion in the lattice with no effect on the fcc ordering of the O sublattice.

In Supplementary Figures 14 and 15, the AIMD runs were conducted with an initial 160-atom γ-Ga_2O_3 cell with additional 8/12/16 inserted Ni/Au/Ga atoms, which corresponds to 5/7.5/10 at.% of foreign ion excess, respectively. The quantitative analyses shown in Supplementary Figures 14(d)-(e) and 15(d) were done in the same way as that in Fig. 4 of the main text.

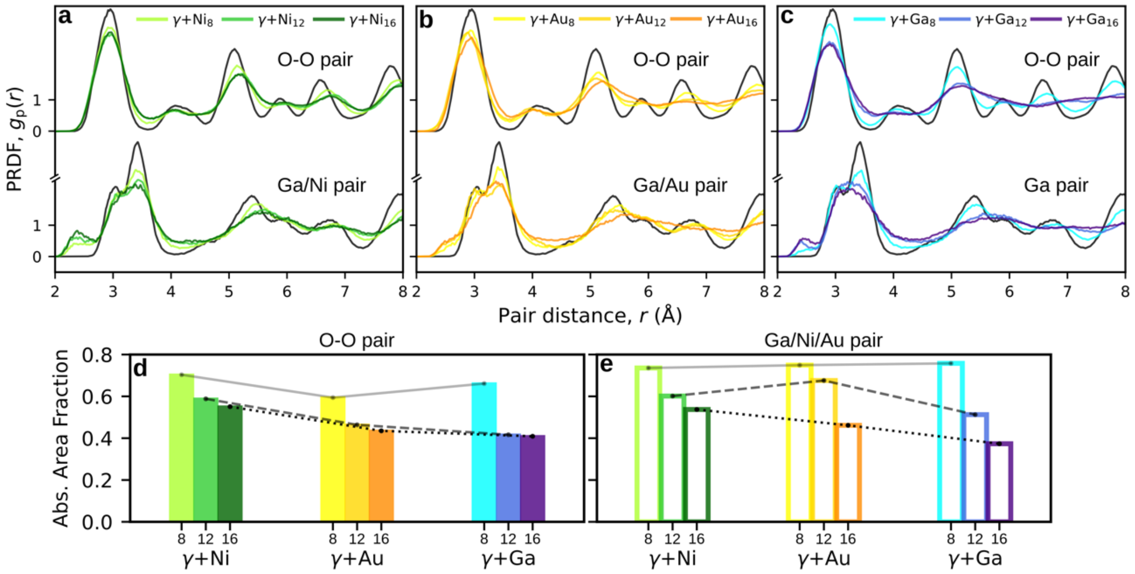

Supplementary Figure 14 (a-c). AIMD-PRDFs of O-O and heavy-ion (Ga/Ni/Au) pairs at 900 K and 0 bar, with different concentrations of the foreign ions. The first valleys are at 3.6 and 4.0 Å, as the same as those labelled in Fig. 4a of the main text. (d, e) Ratios of the absolute areas (covered by the PRDF curves with reference to 1): the distorted cells against the initial γ cell.

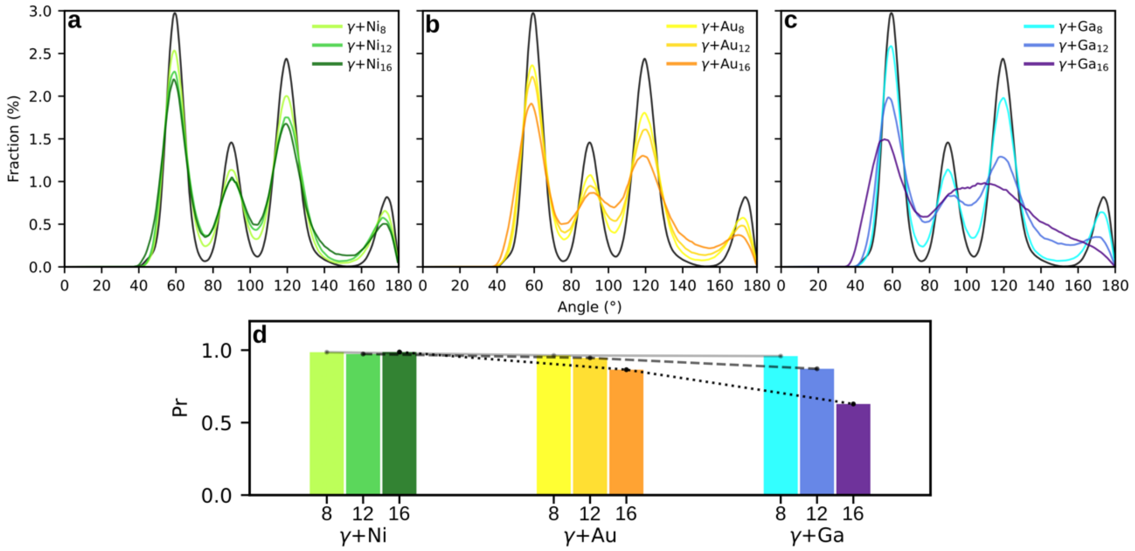

Supplementary Figure 15 (a-c). Bond angle distribution of O sublattice with O-O bond cutoff at 3.6 Å, with different concentrations of the foreign ions. The black curves are the pristine ones without the foreign ions. (d) The Pr values of the distorted bond-angle distribution to the one of the initial γ cell.

Supplementary References:

1. A. I. Titov, A. Yu. Azarov, L. M. Nikulina, and S. O. Kucheyev, "Damage buildup and the molecular effect in Si bombarded with PFn cluster ions", Nucl. Instrum. Methods Phys. Res. B **256**, 207 (2007).

2. J. F. Ziegler, M. D. Ziegler, and J. P. Biersack, "SRIM—the stopping and range of ions in matter (2010)", Nucl. Instrum. Methods Phys. Res. B **268**, 1818 (2010**)**.

3. γ-Ga_2O_3 Crystal Structure. Inorganic Solid Phases, SpringerMaterials (online database), Heidelberg (ed.) (https://materials.springer.com/isp/crystallographic/docs/sd_1201199).

4. B. R. Tuttle, N. J. Karom, A. O'Hara, R. D. Schrimpf, and S. T. Pantelides, "Atomic-displacement threshold energies and defect generation in irradiated β-Ga_2O_3: A first-principles investigation", J. Appl. Phys. **133**, 015703 (2023).

5. E. Wendler, E. Treiber, J. Baldauf, S. Wolf, and C. Ronning, "High-level damage saturation below amorphisation in ion implanted β-Ga_2O_3", Nucl. Instrum. Methods Phys. Res. B **379**, 85 (2016).

6. K. Lorenz, M. Peres, M. Felizardo, J. G. Correia, L. C. Alves, E. Alves, I. López, E. Nogales, B. Méndez, J. Piqueras, M. B. Barbosa, J. P. Araújo, J. N. Gonçalves, J. Rodrigues, L. Rino, T. Monteiro, E. G. Víllora, K. Shimamura, "Doping of Ga_2O_3 bulk crystals and NWs by ion implantation", Proc. SPIE **8987**, 89870M (2014).

7. A. Azarov, C. Bazioti, V. Venkatachalapathy, P. Vajeeston, E. Monakhov, and A. Kuznetsov, "Disorder-induced ordering in gallium oxide polymorphs", Phys. Rev. Lett. **128**, 015704 (2022).

8. E. A. Anber, D. Foley, A. C. Lang, J. Nathaniel, J. L. Hart, M. J. Tadjer, K. D. Hobart, S. Pearton, and M. L. Taheri, "Structural transition and recovery of Ge implanted β-Ga_2O_3", Appl. Phys. Lett. **117**, 152101 (2020).

9. J. García-Fernández, S. B. Kjeldby, P. D. Nguyen, O. B. Karlsen, L. Vines, and Ш. Prytz, " Formation of γ-Ga_2O_3 by ion implantation: Polymorphic phase transformation of β-Ga_2O_3" Appl. Phys. Lett. **121**, 191601 (2022).

10. T. Yoo, X. Xia, F. Ren, A. Jacobs, M. J. Tadjer, S. Pearton, and H. Kim, "Atomic-scale characterization of structural damage and recovery in Sn ion-implanted β-Ga_2O_3", Appl. Phys. Lett. **121**, 072111 (2022).

11. A. Azarov, V. Venkatachalapathy, I.-H. Lee, and A. Kuznetsov, "Thermal versus radiation-assisted defect annealing in β-Ga_2O_3", J. Vac. Sci. Technol. A **41**, 023101 (2023).

12. M. Heczk, D.Esser, T. M. Smith, P. Beran, V. Mazánová, D. W. McComb, T. Kruml, J. Polák, and M. J. Mills, "Atomic resolution characterization of strengthening nanoparticles in a new high-temperature-capable 43Fe-25Ni-22.5Cr austenitic stainless steel", Mater. Sci. Eng. A **719**, 49 (2018).

13. Y. Wang and W. Zhang, "Mapping the strain distribution within embedded nanoparticles via geometrical phase analysis", Micron **125**, 102715 (2019).

14. Ø. Prytz, J. Taftø, C.C. Ahn, and B. Fultz, "Transition metal d-band occupancy in skutterudites studied by electron energy-loss spectroscopy", Phys. Rev. B **75**, 125109 (2007).

15. G. Radtke. M. Hennes, M. Bugnet, Q. M. Ramasse, X. Weng, D. Demaille, B. Gobaut, P. Ohresser, E. Otero, F. Choueikani, A. Juhin, P. Sainctavit, Y. Zheng, and F. Vidal, "Atomic-scale study of metal–oxide interfaces and magnetoelastic coupling in self-assembled epitaxial vertically aligned magnetic nanocomposites", Adv. Mater. Interfaces **6**, 1900549 (2019).

16. H. Y. Playford, A. C. Hannon, E. R. Barney, and R. I. Walton, "Structures of Uncharacterised Polymorphs of Gallium Oxide from Total Neutron Diffraction", Chem. - Eur. J. **19**, 2803 (2013).

17. J. Ahman, G. Svensson, and J. Albertsson, "A reinvestigation of β-gallium oxide", Acta Crystallogr., Sect. C: Cryst. Struct. Commun. **52**, 1336 (1996).

18. A. Azarov, V. Venkatachalapathy, P. Karaseov, A. Titov, K. Karabeshkin, A. Struchkov, and A. Kuznetsov, "Interplay of the disorder and strain in gallium oxide", Sci. Rep. **12**, 15366 (2022).

19. P. Chekhonin, J. Engelmann, C.-G. Oertel, B. Holzapfel, and W. Skrotzki, "Relative angular precision in electron backscatter diffraction: A comparison between cross correlation and Hough transform based analysis", Cryst. Res. Technol. **49**, 435 (2014).

20. L. E. Ratcliff, T. Oshima, F. Nippert, B. M. Janzen, E. Kluth, R. Goldhahn, M. Feneberg, P. Mazzolini, O. Bierwagen, C. Wouters, M. Nofal, M. Albrecht, J. E. N. Swallow, L. A. H. Jones, P. K. Thakur, T.-L. Lee, C. Kalha, C. Schlueter, T. D. Veal, J. B. Varley, M. R. Wagner, and A. Regoutz, "Tackling Disorder in γ-Ga_2O_3", Adv. Mater. **34**, 2204217 (2022).

21. S. Zaefferer, "On the formation mechanisms, spatial resolution and intensity of backscatter Kikuchi patterns", Ultramicroscopy **107**, 254 (2007).

22. K. Schmid, "Some new aspects for the evaluation of disorder profiles in silicon by backscattering", Radiat. Eff. **17**, 201 (1973).
